# Insights from the scale-up and implementation of the Deadly Liver Mob program across nine sites in New South Wales, Australia, according to the RE-AIM framework

**DOI:** 10.1186/s12954-023-00889-5

**Published:** 2023-10-20

**Authors:** Elena Cama, Kim Beadman, Mitch Beadman, Melinda Walker, Carla Treloar

**Affiliations:** Centre for Social Research in Health, John Goodsell Building, UNSW Sydney, Sydney, NSW 2052 Australia

**Keywords:** Aboriginal and Torres Strait Islander people, Blood borne viruses, Health promotion, Hepatitis C, Sexually transmissible infections

## Abstract

**Background:**

The Deadly Liver Mob (DLM) program is a peer-led health promotion program that aims to improve access to screening and treatment for blood borne viruses and sexually transmissible infections for Aboriginal and Torres Strait Islander Australians. In this paper, we used client and staff insights to explore the successes and challenges of implementing the DLM program according to the RE-AIM framework, which explores real-world implementation of interventions according to reach, effectiveness, adoption, implementation, and maintenance.

**Methods:**

Clients and staff were recruited through the DLM program. Semi-structured interviews were conducted with four Aboriginal and Torres Strait Islander and 11 non-Aboriginal or Torres Strait Islander health workers, as well as 33 Aboriginal and Torres Strait Islander clients of the program.

**Results:**

Findings show the positive effects of the DLM program, in creating a culturally safe and sensitive environment for Aboriginal and Torres Strait Islander clients to access care. In particular, the employment of frontline Aboriginal and Torres Strait Islander workers to deliver the education was touted as one of the primary successes of the program, in enabling workers to build trust between clients and mainstream health systems, which has the flow on effect of encouraging clients to go through to screening. The use of the RE-AIM framework illustrates the challenges of implementing real-world interventions across various locations, such as the difficulties in delivering DLM in regional and remote areas due to covering large geographic areas with minimal public transport available.

**Conclusions:**

The data emphasise the need for interventions to be adaptable and flexible, altering elements of the program to suit local and community needs, such as by offering mobile and outreach services to enable access across regional and rural areas. The findings of this evaluation have been used to develop tools so that the learnings from DLM can be shared with others who may be hoping to implement DLM or other similar programs.

## Introduction

This paper presents on qualitative data from a mixed-methods evaluation of the Deadly Liver Mob (DLM) program, a peer-driven, incentivised health promotion program, which first began as two pilot sites in 2013 and 2015 in New South Wales, Australia. The aim of the program is to provide a culturally safe, sensitive, and appropriate way to increase access of Aboriginal and Torres Strait Islander peoples to testing and treatment for blood borne viruses (BBVs) and sexually transmissible infections (STIs) at mainstream needle and syringe programs (NSP) and sexual health services. First, we provide a review of the data on rates of BBVs and STIs among Aboriginal and Torres Strait Islander communities, which provides the background context and rationale for the establishment of the DLM program. We then provide an overview of the DLM program model, before turning to the methods and findings from qualitative interviews with DLM clients and staff.

### Background context on BBVs and STIs among Aboriginal and Torres Strait Islander communities

Census data indicate that in 2021 there were approximately 812,728 Aboriginal and Torres Strait Islander people in Australia, representing 3.2% of the total population [1]. However, significant disparities in notification rates for BBVs and STIs exist for Aboriginal and Torres Strait Islanders compared to non-Aboriginal or Torres Strait Islander Australians. Surveillance data suggest that notification rates per 100,000 for BBVs among Aboriginal and Torres Strait Islander people including HBV, HCV, and HIV^1^ are reported as being as much as 1.8, 5.9 and 1.6 times higher compared to non-Aboriginal or Torres Strait Islander Australians [2, 3]. For STIs, such as chlamydia, gonorrhoea, and syphilis, these rates (per 100,000) have been reported at 2.8, 4.2, and 5.5 times the rate for Aboriginal and Torres Strait Islander compared to non-Aboriginal or Torres Strait Islander people [3]. An ongoing infectious syphilis outbreak has resulted in significant increases in notification rates among Aboriginal and Torres Strait Islander communities [4]. Data also indicate that Aboriginal and Torres Strait Islander people living in remote and very remote regions of Australia are disproportionately impacted by BBVs and STIs [2, 3].

Ward and colleagues [5] argue that STI risk among Aboriginal and Torres Strait Islander communities must be understood in the context of a range of factors. These include earlier age of sexual debut and number of sexual partners [6], but also include a range of social determinants impacting on Aboriginal and Torres Strait Islander people, including access to healthcare, poverty, access to education, social disadvantage, among others [7]. Access to and barriers to screening for BBVs and STIs also remain important considerations. Given that BBVs and STIs may be asymptomatic, screening in the context of primary health services is largely opportunistic. Historically there has been low rates of participation of Aboriginal and Torres Strait Islander communities within primary health services [8]. This must be viewed in the context of historical, political, social, and cultural determinants of health which impact on the health of and access to health care for Aboriginal and Torres Strait Islander communities, including the history of colonisation, socio-economic disadvantage, cultural identity, and self-determination [9, 10].

Other individual and systemic barriers that may impede the access of Aboriginal and Torres Strait Islander peoples to primary health services include limited knowledge about BBVs and STIs, limited skills among health providers to effectively communicate with Aboriginal and Torres Strait Islander people about BBVs and STIs,^2^ concerns about confidentiality, mistrust in non-Aboriginal or Torres Strait Islander health workers and health systems, shame and stigma around seeking access to primary care for sexual health, prohibitive health care costs, inappropriate design (e.g., not separating genders according to Aboriginal and Torres Strait Islander cultural norms) and location of health services, high staff turnover and limited health services available across large geographic areas in rural and remote areas, limited transportation access, lack of culturally appropriate and sensitive health care, and institutional racism resulting in, for example, longer wait times to receive care [11–19]. Taken together, these data highlight the need to improve access to BBV and STI screening through the development of culturally appropriate and safe health services, which can overcome some of the barriers outlined above.

### The DLM model

The DLM program was introduced in 2013 as a pilot program in one publicly funded NSP, followed by a second pilot site in 2015. The program aimed to overcome systemic barriers to mainstream health care participation faced by Aboriginal and Torres Strait Islander communities by providing a culturally safe, sensitive, and appropriate pathway to engagement in the service. The DLM program is modelled from a program known as the Safe Injecting Cwiz (SIC), which was implemented in Western Sydney, New South Wales between 1998–2002, targeting people who injected drugs and were under the age of 25 years [20]. In turn, the SIC program was adapted from a U.S. based HIV peer-driven intervention, which sought to reach hidden networks of people who inject drugs [21–23].

The DLM program is for Aboriginal and Torres Strait Islander people, and Aboriginality is determined by the local Aboriginal and Torres Strait Islander DLM workers and in accordance with community principles. Additional eligibility criteria for the DLM program are that clients must have ever or currently inject drugs OR are classified as ‘at risk’ for injecting drug use, BBVs, or STIs. ‘At risk’ in the program is defined as people who have a prison history, unsafe tattoo, or that they are living with a person/people who inject drugs or who have/have had HCV. Aboriginal and Torres Strait Islander clients who enter the program are educated about viral hepatitis then offered screening and any relevant treatment for BBVs and STIs, as well as vaccination for hepatitis A (HAV) or hepatitis B (HBV). The program is unique in that it was developed, implemented, and evaluated in partnership with Aboriginal and Torres Strait Islander people, for Aboriginal and Torres Strait Islander people.

At each stage of the program, clients are offered nominal incentives: education, recruitment of peers, screening, treatment for STIs, and vaccination for HAV and HBV (see [24] for program logic; [25]). Clients diagnosed with HCV are referred to HCV treatment, but do not receive incentives for such treatment. The two pilot sites commenced prior to the introduction of direct-acting antiviral (DAAs) therapy for HCV and offer refresher education to returning clients to provide the most up-to-date treatment information. Non-Aboriginal or Torres Strait Islander partners of eligible clients are permitted to enter the program, given the importance of extending BBV and STI screening to sexual partners [26–28], but do not receive the incentive vouchers and are not recorded as part of the evaluation.

Evaluation of the two early pilot sites showed increases in Aboriginal and Torres Strait Islander clients attending the mainstream health services, as well as evidence of high acceptability of the program among clients and staff [24]. Following the success of the pilot sites, funding was received through a National Health and Medical Research Council Partnership Grant and the NSW Ministry of Health to scale-up, implement, and evaluate the DLM program at an additional seven sites, bringing the total DLM sites to nine within seven Local Health Districts (LHDs)^3^ in New South Wales. During this evaluation there were nine DLM sites within seven LHDs: three are located in the Sydney metropolitan area and six in regional and rural New South Wales (NSW). While the program initially was designed to operate out of NSPs and sexual health services, sites have since broadened the scope to include outreach, such as to community health centres.

This paper presents findings from qualitative interviews with both DLM staff and clients to explore the successes and challenges of implementing the DLM program across the nine sites. We focus specifically on staff and client insights about the DLM program as they apply to each of the components of the RE-AIM framework, which is defined in the Methods section.

## Methods

The evaluation of the DLM program is mixed-methods and uses insights from the RE-AIM model [29], which consists of five dimensions to guide evaluations of real-world implementation models: Reach, Effectiveness, Adoption, Implementation, and Maintenance. The five dimensions and how they apply to the DLM evaluation framework are described elsewhere [25] but will be briefly outlined here. Reach refers to the number of people who participate in the intervention and how representative they are of the population; Effectiveness refers to the impact of the intervention on the outcomes of interests, as well as any potential negative effects of the intervention; Adoption is the number and representativeness of the settings where the intervention is implemented, as well as those who deliver the intervention; Implementation refers to the extent to which the program was delivered as it was originally designed and intended and can include a range of individual and organisational factors that contribute to its success; and Maintenance is the sustainability of the intervention, or the extent to which the intervention becomes a routine part of the institution [29, 30].

This paper reports on data from interviews with clients and staff of the DLM program, drawn from a broader evaluation of the program conducted by the Centre for Social Research in Health at UNSW Sydney. More broadly, the DLM program and the associated mixed-methods evaluation are designed to be low threshold, with minimal client data collected in order to remove any potential barriers to Aboriginal and Torres Strait Islander clients engaging in the program. This means that, with the exception of the interviews, clients are not required to individually consent in order for the researchers to access data. However, data collected by the researchers where individual consent is not collected is that which is routinely collected by the service and is de-identified. The waiver of consent approach and all other aspects of the evaluation were approved by the South Eastern Sydney LHD Ethics Committee and the Aboriginal Health and Medical Research Council Ethics Committee. Site-specific approvals were also obtained from the LHDs governing each of the DLM sites.

Aboriginal and Torres Strait Islander clients were invited to take part in semi-structured interviews to speak more about their experiences of the DLM program. Interviews were conducted by Aboriginal researchers (MW, MB, KB) employed at the Centre for Social Research in Health, UNSW Sydney. Consent for interviews was obtained via telephone and clients were provided with a voucher to compensate them for their time. Interviews were audio-recorded and transcribed by a professional transcriber operating under a confidentiality agreement.

Health staff who were involved in the development and implementation of the DLM program were also invited to take part in semi-structured interviews to speak about their experiences of the scale-up and implementation. Staff who were interviewed included frontline Aboriginal and Torres Strait Islander workers, program managers, and sexual health clinicians. Interviews with Aboriginal and Torres Strait Islander staff were conducted by Aboriginal researchers (MW, MB, KB), while interviews with non-Aboriginal or Torres Strait Islander staff were conducted by a non-Aboriginal or Torres Strait Islander staff member from the Centre for Social Research in Health, UNSW Sydney. Staff were not provided with vouchers or monetary compensation. Interviews were audio-recorded and transcribed by the same professional transcriber as the client interviews.

All potentially identifying information about staff and clients were removed from the transcripts. We analysed the transcripts in relation to the components of the RE-AIM framework, which are Reach, Effectiveness, Adoption, Implementation, and Maintenance. We were interested in exploring the perspectives of health staff and clients about the expansion of the program to new sites, including the ability to implement the program as intended, the positive and negative impacts of the program, and whether the program could be absorbed within the site as part of routine everyday practice. Following initial data analyses by the first author (EC), analyses and interpretation were workshopped with Aboriginal (KB, MB) and non-Aboriginal (CT, EC) authors. We only identify participants according to whether they are Aboriginal and Torres Strait Islander or non-Aboriginal or Torres Strait Islander, and staff or clients, due to the risk of identifying participants if we were to provide further information.

## Results and discussion

The sample consisted of four Aboriginal and Torres Strait Islander and 11 non-Aboriginal or Torres Strait Islander health workers, as well as 33 Aboriginal and Torres Strait Islander clients of DLM. Health workers were involved in various elements of DLM, such as the development and implementation of the program, and delivery of the program, such as Aboriginal and Torres Strait Islander frontline workers and clinical staff providing sexual health screening.

### Reach

In relation to reach, we were interested in the ability of the DLM program to bring clients into the program. Elsewhere, we have outlined the numbers of clients entering and progressing through the DLM program [31], and here we focused on worker and client perspectives on the factors that facilitated or acted as a barrier to client engagement. The key points that were raised by staff and clients in respect to aspects of the program that encouraged clients to enter the program were the culturally appropriate and safe environment, through employment of a frontline Aboriginal or Torres Strait Islander worker and the incentive vouchers. The key challenges described by workers and clients were following up clients and the ability to implement and deliver the program, particularly in rural and remote areas, given the limited locations that ran DLM and the challenges in promoting the program.

A culturally appropriate or safe program would be one that develops trust and respect between health systems, health workers, and Aboriginal and Torres Strait Islander peoples [32]. To improve health care accessibility and ensure that care provided is culturally appropriate and safe, employing Aboriginal and Torres Strait Islander health staff and ensuring community stewardship are critical [12, 33, 34]. Other researchers have also emphasised the important and unique skills and values that Aboriginal and Torres Strait Islander people bring to the health workforce [35], as well as the benefits of partnerships between Aboriginal and mainstream health services [36]. Elsewhere, we have described how designing the program in partnership with Aboriginal and Torres Strait Islander people and having Aboriginal and Torres Strait Islander frontline workers as part of the DLM pilot sites was touted as one of the primary factors for its success in meeting community needs [24]. With the scale-up and implementation of the program at an additional seven sites, the ongoing partnership with and employment of Aboriginal and Torres Strait Islander workers to be the face of DLM were seen as critical to engaging Aboriginal and Torres Strait Islander communities. Staff described that Aboriginal and Torres Strait Islander workers were able to get the word out about the program through their established links with community and to build trust between Aboriginal and Torres Strait Islander clients and mainstream health systems.

It’s about your clients and the community and feeling safe and comfortable, to suss you out, know that they have come to talk to you on other things and they might do that, and then once they know that they feel safe and comfortable with you, then they’re more likely to go through the education and maybe even screening. (Aboriginal or Torres Strait Islander DLM worker)

The expectation is to be able to get as many Aboriginal mob coming through the door so that they can be screened and hopefully prevent like preventable illnesses and for us to be spreading the word in the community and accept within the community that it’s a safe place to come and have a yarn about their business. (Aboriginal or Torres Strait Islander DLM worker)

Clients described that staff were friendly and non-judgemental, providing a safe space for them to discuss health concerns and injecting drug use history. As one client said, ‘Because they are nice to you and they don’t judge you and they just like tell you as it is sort of thing.’ (DLM client). Although this was not logistically possible at all sites, those sites that were able to employ both a male and female Aboriginal or Torres Strait Islander worker were seen as best practice in accordance with community principles.

We’ve got both a male and female Aboriginal sexual health worker and I believe having both was beneficial for the education and, you know, progressing people into screening following that education… I think it stems back to again in [this community], a bit of the men’s business, women’s business. (Aboriginal or Torres Strait Islander DLM worker)

The DLM program provides clients with incentives to progress through the program, from education, to screening, returning for results, and receiving any required HAV or HBV vaccination or treatment for STIs. Financial incentives have been shown by previous research to be effective in altering health behaviours and increasing uptake of health interventions, particularly in low- and middle-income countries [37–39]. Although not considered to be standard practice in the Australian context, there is evidence to suggest that the use of incentives can increase uptake of STI screening [40–42]. Staff and clients described that the incentives enabled clients to enter the program, citing additional benefits of clients being able to buy food, which was particularly helpful for those who could not otherwise afford these purchases. As one client said, ‘a lot of people I suspect are in my position that you can’t afford fresh veg [vegetables] and fruit and good stuff for yourself that you need and they [the vouchers] come in so handy.’ (DLM client). Clients believed that the DLM would not otherwise be able to reach clients, with the incentives touted as the impetus to enter the program. As the third excerpt below highlights, the incentives were a ‘draw card’, with clients referring other people to the program to also receive these vouchers.

I think if you didn’t have the vouchers, no one would pull up and have the time to check themselves, you know. Yeah, that’s what I reckon, so it’s a good thing, because that will push them to come along. (DLM client)

I reckon it’s a great incentive, the best one. It does encourage people you know, not only to get themselves sorted out, they can get a little bit of tucker [food] with the vouchers too and they can walk away and go, “I might have had a blood test, but I’m eating well tonight”. Some people can’t live right, eat right or can’t budget their money right and that voucher there, gives them that incentive to buy a bit of tucker. (DLM client)

Vouchers are a big draw card. Some places were good, because some of the outreach places we worked at, once someone came in that knew about the vouchers and that, they went out and grabbed other people who came in and then they went out and grabbed more people, so that was a really good thing. (Aboriginal or Torres Strait Islander DLM worker)

Health systems must overcome individual and systemic barriers to health care access for Aboriginal and Torres Strait Islander Australians (e.g., see [12, 14, 16–18]). This presented challenges for health workers, such as in following up clients and in the ability to deliver and promote the program, particularly in regional and remote areas. For following up clients, DLM workers described that there were challenges in doing so to ensure clients progressed through the program (such as to receive results of screening), due to phone numbers changing or, in some instances, clients passing through the area. With permission from the client, some sites tried to overcome this barrier by asking other services to let the client know to come back to the clinic if they saw them at the service.

[W]hat didn’t work was when trying to get them back for … because their phone numbers change and stuff like that, so it was trying to get them back for their results or they come in for their results. (Aboriginal or Torres Strait Islander DLM worker)

Many people we see don’t have telephones… that can be quite hard and a challenge for us [to follow up]. (Non-Aboriginal or Torres Strait Islander DLM worker)

The second key barrier for reach was the ability to deliver and promote the program in regional and remote areas. For instance, in regional and remote areas, there may only be one site and travel distances to the site might be significant, which presented a barrier to clients if they did not have transport to the service. As one Aboriginal or Torres Strait Islander DLM worker described, ‘if they didn’t have transport, how could they actually get down to the venue to see me? So, there are a lot of struggles in terms of where people live and what sort of transportation they have and being out in the [remote area], even public transport is just almost non-existent.’ As will be described later, some sites ran outreach into regional and remote areas in order to increase the reach of the program, although this can come with its own challenges (such as ensuring clients are provided with results from BBV and STI screening). Some clients also believed that DLM should be run in multiple locations, to provide clients with options for where to attend. They also believed that running the program in high density sites, for instance close to Centrelink locations (the government office for social security payments and services), would improve the reach of the program. Further, clients suggested that greater advertising could be done to more widely promote the program.

Getting it out there more, yeah more advertisement so that if something does come up, I tend to let people know and really I’d like to see more of them, yeah. It’s a good little thing. (DLM client)

[I]t could be in other places, because some people don’t access this centre… I guess having various locations, because you know a place this big… So it would be good to have various locations within a place, more central where people have got to go in and do their business elsewhere could be you know close by for example like a Centrelink place maybe or a place where people go for you know business, their own business, shopping maybe. (DLM client)

### Effectiveness

The DLM program aims to increase access to testing and treatment of BBVs and STIs, including provision of education to clients on topics such as HCV and injecting equipment sharing. Elsewhere, we have described data on clients’ progression through the stages of the program [31]. In interviews, both staff and clients suggested that the DLM program was effective in achieving its stated aims. Some clients had previously had minimal engagement with mainstream health services due to historically negative interactions, and the historical, political, cultural, and social determinants of health and health care access [9, 10]. As one Aboriginal or Torres Strait Islander DLM worker said, ‘Some of them have actually lost faith in health services.’ The DLM program thus provided an entry point for Aboriginal and Torres Strait Islander clients into these services. As the first excerpt below highlights, one client described the program as providing them with the confidence to have testing and treatment for HCV. In the second excerpt, an Aboriginal or Torres Strait Islander worker described the importance for frontline workers to firstly build trust between clients and mainstream systems, which then lead clients to feel safe and comfortable to go through the stages of the program i.e., screening.

I thought it was really good that they were educating people on how you can catch it and what you could catch it from and how you can treat it and where you can get it treated… So they gave me the confidence to be able to go and get treated and go and get the tests done and stuff like that and the liver function test and everything, so yeah. (DLM client)

I think they are very nice, very open minded, never judgmental and they make you feel comfortable… And that’s what keeps me coming back and I feel comfortable with this and even just getting the blood taken, when I got the blood taken, that’s a big thing and just having that atmosphere in the room at the time, yeah, it’s a lot smoothing and helpful. (DLM client)

It’s about your clients and the community and feeling safe and comfortable, to suss you out, know that they have come to talk to you on other things and they might do that, and then once they know that they feel safe and comfortable with you, then they’re more likely to go through the education and maybe even screening. (Aboriginal or Torres Strait Islander DLM worker)

While the focus of DLM is specifically on BBVs and STIs, Aboriginal and Torres Strait Islander workers described an additional benefit of their presence, which was that it could allow clients to speak about other health issues that they were facing. For sites with good connections with other mainstream health and social services, there were good referral pathways to ensure that clients could address these other needs. Due to political, cultural, and social determinants of health, Aboriginal and Torres Strait Islander communities face significant health disparities, including high prevalence of chronic health conditions such as diabetes, heart and liver disease, and cancer, contributing to a gap in mortality among Aboriginal and Torres Strait Islander and non-Aboriginal or Torres Strait Islander Australians [43]. Thus, the DLM program presents a unique opportunity to facilitate referral pathways to other health and social services. A literature review of Aboriginal and Torres Strait Islander peoples engagement in the health workforce referred to the vital role that the Aboriginal and Torres Strait Islander health workers plays in acting as ‘cultural brokers’, supporting access to mainstream health care [44]. In particular, the review identified that while non-Aboriginal or Torres Strait Islander health workers are trained to enforce professional boundaries with clients, Aboriginal and Torres Strait Islander health staff use ‘connectedness and shared experience… and see the care they provide as part of their broader relationship’ [44]. However, the benefits of the Aboriginal and Torres Strait Islander health workforce must be considered in the context of the challenges in responding to multiple demands and being expected to take on multiple roles (e.g., social worker, community advocate, counsellor) [44]. In interviews about DLM, this was apparent in descriptions of potential burnout of the frontline Aboriginal and Torres Strait Islander workers, who sometimes described wanting to do more to help their community but feeling overwhelmed. As one said, ‘you do get a little overloaded. You try to do what you can.’ (Aboriginal or Torres Strait Islander DLM worker). This finding suggests a need for greater support of designated Aboriginal and Torres Strait Islander workers within health services.

I think having an Aboriginal person who is safe and comfortable without feeling pressured or judged or anything else, to be able to talk about stuff… The client will come up and once they start feeling comfortable with you, they are going to start talking about everything else and then before you know it, you actually try to work with them on all the other issues that are not yours, particularly in like that sort of community, being in a far, you know, what they call a rural/remote community… You try to do what you can, you try to make phone calls and maybe take them to the other service as a one-off to help build that relationship with that other service… We see it at a holistic level and if we can tap in and try to put that person to access those other services that are required. (Aboriginal or Torres Strait Islander DLM worker).

The provision of incentives for participation in health programs is potentially divisive for some health staff. In the pilot program, some staff expressed concerns about the use of vouchers as ‘bribing’ clients to undergo education and screening [24]. Following the scale-up of the program across additional sites, the general consensus was that the incentives were a positive and major contributor to the success of the program, not just in terms of the initial reach, but in encouraging clients to progress through the stages of the program. Staff still described that people outside of DLM may have negative opinions about providing Aboriginal and Torres Strait Islander clients with incentives to participate, but these sentiments were not shared by those involved in the implementation of the program. Instead, incentives were described as essential for getting clients into the service, resulting in increased presentations and access to screening. Providing incentives could result in a positive experience for the client with health systems in which historically they may have had negative experiences, thus providing encouragement to access these health services in future. Incentives are provided for clients to receive treatment for STIs and HAV and HBV vaccination but are not provided for engagement in treatment for HCV. Thus, some workers suggested that sites should explore funding such treatment moving forward, given that increasing access to treatment for HCV is one of the priorities of the program.

I think it’s great. I mean whatever works really. That’s my overall impression is, is whatever works, works and you know some people might say that giving money is not the right way to go about things, but I disagree, I think if they can come in and they get a good meal that otherwise wouldn’t, then that’s a positive experience that they relate to health care in itself. (Non-Aboriginal or Torres Strait Islander DLM worker)

Although incentives were considered to play an important role for getting clients into the service, staff also described that Aboriginal and Torres Strait Islander clients wanted to be involved irrespective of the incentives once word spread about the program. Thus, incentives were just a ‘bonus’ (Non-Aboriginal or Torres Strait Islander DLM worker) to clients becoming involved. Further, clients remained engaged in the program for other reasons beyond incentives, such as the relationships built with Aboriginal and Torres Strait Islander staff and the increased levels of trust in mainstream health system following their experiences with DLM. In particular, clients described coming back due to the relationships established with DLM workers, who were described as supportive and like a friend.

It was more the education [than the incentives] and I think the people who were involved with it at the time, they were just really supportive, really open and more like a friend… Yeah, because they are just really nice, kind people that are kind hearted… Not judgmental at all you know, they will give you a cuppa, they will have a yarn with you. (DLM client)

Definitely the incentives bring people in, but it’s not just … it doesn’t seem to be just the incentive because people want to be screened anyway once they’ve heard about it. I think too, family members that are perhaps Aboriginal and not injecting drug users, but they know somebody, so that attracts them to bring … interest to bring their family members or people they know to come and learn more about hepatitis C and be tested. (Non-Aboriginal or Torres Strait Islander DLM worker)

Where metro sites had great success in seeing early and steep increases in the numbers of Aboriginal and Torres Strait Islander clients accessing the service, these numbers tended to plateau over time. Conversely, progress was slower in rural and regional sites. It’s important to carefully consider how ‘success’ is defined in programs such as DLM given contextual factors [24]: for example, metro sites have higher density populations and greater transport options, thus it is expected that they would see these steeper increases which then plateau. Given the historically low rates of Aboriginal and Torres Strait Islander engagement in mainstream health services, as well as ongoing systemic barriers to health care access that can make engagement of Aboriginal and Torres Strait Islander communities challenging [11, 12, 14–19], any improvement in attendance from baseline (prior to intervention) is deemed to be a success. Therefore, such a plateau (or even decline) in participant numbers should not be the basis for discontinuation of interventions like DLM. Rural and regional sites may not see these sharp increases in client engagement but are likely to see steady numbers over time, while metro sites may see initially sharp increases in client engagement which plateau over time.

[Y]ou get hit hard and fast… and then it’s more of a trickle effect because you have almost plateaued at who you can recruit for that site if that makes sense. (Non-Aboriginal or Torres Strait Islander DLM worker)

[W]e saw lots of clients and they came back and I would say it was very successful, but obviously what we’ve found with time is that in some ways we started to our exhaust those numbers. (Non-Aboriginal or Torres Strait Islander DLM worker)

### Adoption

Adoption is concerned with the settings in which the intervention is implemented, and the people who deliver the intervention [29]. The DLM program has been established in nine sites within seven LHDs. The DLM program was designed to operate out of NSPs and sexual health clinics, however at some sites the sexual health clinic is not as closely co-located to the NSP as at the original pilot sites. At these sites, clients must travel to a nearby hospital for screening for BBVs and STIs. This does present a barrier to client engagement and progression through the various components of the DLM program. However, staff at these sites have trialled various strategies to overcome this barrier, such as by having the DLM worker walk clients over to the hospital to ensure they are appropriately introduced to sexual health staff, many of whom are non-Aboriginal or Torres Strait Islander.

[I]f you don't have an Aboriginal sexual health or Deadly Liver Mob worker directly located near one of the clinics, it can be a little difficult to maintain engagement with the client to get them tested, so the part from recruiting into DLM from NSP to getting them across to the site for testing. (Non-Aboriginal or Torres Strait Islander DLM worker)

Many sites have since expanded the program to include outreach into the community, responding to some of the challenges in reaching clients and to be able to cover greater geographic areas, especially in rural and remote areas of the state. For one site, they repurposed a van held by the service, and used it as part of outreach. As the first excerpt below notes, there is a need to take the DLM program out into the community, given that many people may not be within easy distance of a DLM site. It’s important to note that this frontline staff member suggested a continued need to go out into the community, as they perceived the importance of having a ‘community base’. The second excerpt below highlights a point also made within Effectiveness, which is the potential to reach saturation at some sites, and the need to vary the recruitment methods in order to reach new clients.

[T]he availability to deliver the clinics in a community setting, so from a hub, we have a community hub which is based out in where a lot of the Aboriginal people live in [social] housing, so obviously the need for an Aboriginal worker to go out and access community, so we would run that clinic from that hub, where I would go out, knock on doors, have a yarn and talk about the program and bring them back, … I think what worked well is being able to provide that clinic each week out there, so the mob knew where it was. (Aboriginal or Torres Strait Islander DLM worker)

[T]hey [DLM frontline workers] have done [some sites] to saturation with the occasional trickle you know what I mean, but not worthy of putting resources sitting there all day waiting for one punter a week you know what I mean? So I told them to go mobile to other sites… [We’re] getting really good results, but no doubt will hit saturation points there. Hence why we have decided that the people that we really need to get to are the ones that don't turn up at NSPs, so we think the mobile unit will be you know, will probably enhance access out at communities. (Non-Aboriginal or Torres Strait Islander DLM worker]

Staff also reflected on how they had to be innovative with their recruitment approaches, particularly once the initial increases seen following introduction of DLM began to stabilise over time. This included recruiting through Aboriginal and Torres Strait Islander festivals, men’s groups, and post-release from incarceration. One worker described the program as having ‘portability to enhance access’ (Non-Aboriginal or Torres Strait Islander DLM worker), with potential to move around during NAIDOC (National Aborigines and Islanders Day Observance Committee) week, which celebrates the history and culture of Aboriginal and Torres Strait Islander peoples in Australia. The below excerpts highlight both the strategies that sites had already taken to improve Reach and diversify Adoption, as well as how sites were continuously brainstorming how to do so moving forward.

[W]e had a ground swell of people working there to engage with Aboriginal people who were attending the [Aboriginal and Torres Strait Islander] Festival to engage with them and talk about what we do and there was some dry blood spot testing undertaken on that day and we did some peroxide training so that’s a fantastic initiative that we could do at such a festival. People know us and recognise us and they know what we do, but it’s a way to introduce us to the broader community to promote the health and well-being of all clients. (Non-Aboriginal or Torres Strait Islander DLM worker)

[W]e’re going to have to find new ways of reaching out, so we’re just about to start a DLM program down at [a hospital]… and it’s got a clinical room and once our new worker is on board, we will be looking at having a DLM program down there, maybe twice a month with one of our nurses and we’re also looking at setting up in another NGO down there, so yeah, we’ve got hopes (Non-Aboriginal or Torres Strait Islander DLM worker)

### Implementation

Within the RE-AIM framework, the dimension of Implementation is partly concerned with whether staff deliver the program as originally planned or intended [30]. To maintain some degree of consistency in delivering the program, the education materials from the original pilot sites were shared with the new sites, and two Aboriginal and Torres Strait Islander DLM workers from the pilot sites filmed a video to demonstrate what an education session might look like. Further, a DLM Management Group was set up, hosting meetings every couple of months to facilitate communication between all the sites. These meetings provided an opportunity for sites to share positive outcomes, but also to share challenges and brainstorm solutions based on the learnings from other sites. One Non-Aboriginal or Torres Strait Islander DLM worker described of these meetings, ‘It was incredibly enriching and empowering for us to know of the work that had been undertaken, the solid grounding that people had in community and how successful this approach could be.’

In designing and implementing the DLM program, all sites stressed the need to be flexible and adaptable, and to tailor the program to meet local community needs. This was in recognition that not all materials and strategies would work across all sites, particularly given the geographic spread across metro and regional and rural areas of the state. Thus, sites also needed to use local resources and referrals during education sessions, as well as make alterations to the program in circumstances where the original model was not working well. For example, metro sites were provided with incentives vouchers for mainstream supermarkets, however this was not appropriate for rural areas which did not have a nearby mainstream supermarket. For these areas, vouchers for other stores, such as smaller chain supermarkets, were provided.

Now, another barrier that we sort of had but we managed to work our way around was when we first started talking about the incentives, the [mainstream supermarkets] sort of vouchers were the way we ended up going, but when we started doing more outreach, so again the location, for example, when they started doing the outreach once a month and we were offering the Deadly Liver Mob there, there’s no [mainstream supermarket] out that way, so we had to negotiate with the local [supermarket] to participate in the project. (Non-Aboriginal or Torres Strait Islander DLM worker)

Some sites also altered the incentive amounts that they were providing to clients. Although the overall maximum incentive amount remained the same, these sites provided a lower amount for education and a higher amount for screening and returning for results. This was due to drop off rates in people returning for screening and results for various reasons, such as travel distance (both to and from the site, as well as from the NSP to sexual health clinic), travel costs, and parking availability at the site. As one Aboriginal and Torres Strait Islander DLM worker noted, ‘It’s a very busy little street too during the day time… a lot of people get dropped off and run in and grab their [injecting equipment] really quick, so that’s why they weren’t sticking around.’ Thus, incentive amounts were varied to try to encourage clients to return given the importance of providing clients with screening and results.

I think it’s a worthwhile reason. I know in other areas, they have possibly changed the incentive schedule because, you know, because cost of travel or the distance to travel was so much that the incentive wasn’t worth it for people. So, they’ve tried to make the incentive more of an incentive, you know what I mean, so that people will come back. (Non-Aboriginal or Torres Strait Islander DLM worker)

### Maintenance

The dimension of Maintenance is largely related to the sustainability of the intervention. This was a core concern for the DLM program, as the grant scheme for the evaluation funded the incentive vouchers for some of the sites for the duration of the evaluation period. Thus, there were concerns about the sustainability of the model beyond the life of the evaluation, when such funding would no longer be available. Staff suggested that services would need to find ways to sustain the incentive vouchers beyond the evaluation, and within their regular budget. It’s important to note that some sites covered the costs for incentive vouchers throughout the evaluation, and thus this aspect of sustainability was only of concern to those sites who had received funding assistance as part of the evaluation.

Probably the only difficulty for us is trying to sustain the gift vouchers. If we could somehow work out a way to sustain the gift voucher costs within our budget, I can see absolutely no reason why DLM shouldn’t keep continuing…. [The funding is] all coming out of this research program, but once the research is completed that would be something that I think we will have to build in and work out how to build that in. The other thing with DLM I think you really need to have Aboriginal workers… to be able to do it. (Non-Aboriginal or Torres Strait Islander DLM worker)

There were additional concerns about staffing, including recruitment, retention, as well as securing ongoing funding for a designated Aboriginal or Torres Strait Islander worker. For Aboriginal and Torres Strait Islander workers, they described the need for more identified Aboriginal and Torres Strait Islander positions, as well as permanent roles rather than short-term contracts. For instance, one Aboriginal or Torres Strait Islander worker described at the time of the interview being unsure whether their contract would be extended and whether they would have a job at that time. Despite Aboriginal and Torres Strait Islander people being a priority population within the national and state BBV and STI strategies [45–48], and more broadly within health, it was apparent that the funding was just not available to secure longer term positions for these workers. This resulted in a small workforce of Aboriginal or Torres Strait Islander health workers, which restricted the capacity of DLM and other programs and what they could achieve. Although the number of Aboriginal and Torres Strait Islander people in the health workforce has increased over time, there has been no real improvement in the proportion of the total workforce, due to the simultaneously increasing number of non-Aboriginal or Torres Strait Islander people in the workforce [35]. Further, a review of the literature identified that Aboriginal and Torres Strait Islander health workers face job insecurity and inequality, due to short-term contracts and inadequate remuneration (particularly when compared to the non-Aboriginal or Torres Strait Islander health workforce), resulting in high staff turnover and loss of the skills that these workers bring to provision of care [44]. Thus strengthening recruitment and retention of Aboriginal and Torres Strait Islander people to the health workforce has been noted as a broader need to ensure culturally appropriate and safe care across chronic health issues [34] and in recognition of the unique and important skills and values that Aboriginal and Torres Strait Islander people bring to the health workforce [35].

I think the need is much greater than what we are able to meet at the moment… We know that all the area health services have limited budgets and yet Aboriginal health continues to be a priority both under Federal and State Governments, but still the money is just not being made available to demonstrate that they think it’s a priority. (Non-Aboriginal or Torres Strait Islander DLM worker)

Throughout the evaluation, there were also disruptions to the implementation of the program due to staff changes or staff unavailability. To overcome some of these issues, in very rare cases, some sites had a non-Aboriginal or Torres Strait Islander staff member cover the DLM program. However, this was only to cover if the Aboriginal or Torres Strait Islander DLM worker was unavailable on the day that the DLM program was run and did not apply to circumstances where the staff member took a longer-term absence or had left the position. In instances where the DLM worker had left the position, DLM was not operational until a new Aboriginal or Torres Strait Islander worker was recruited to the position, which in some cases led to months where the program did not run. These disruptions were further exacerbated by the COVID pandemic, where DLM did not run for several months in any of the sites, particularly in 2021 when outbreaks extended to Aboriginal and Torres Strait Islander communities and staff were required to assist in COVID-related activities.

We’ve had some staff changes over the time with the Aboriginal position… it’s tricky, it’s a reasonably small workforce that we can tap into and because we are also looking for somebody with project management skills as well as direct client skills. (Non-Aboriginal or Torres Strait Islander DLM worker)

Given many of the challenges faced by DLM, which have been detailed in the preceding sections, staff stressed the importance of the DLM model being adaptable and flexible. For instance, Aboriginal and Torres Strait Islanders described how every community is different, and the program may need to be adapted to meet the needs of each local community. The benefit of the program was the ability for it to be mobile, with staff able to conduct outreach into communities. However, Aboriginal and Torres Strait Islander workers were cognisant that much more could be done. Both Aboriginal and Torres Strait Islander and non-Aboriginal or Torres Strait Islander staff indicated that more could be done to address BBVs and STIs in communities, such as by undertaking dried blood spot testing (and ensuring that the frontline staff were trained to do so), advertising the program more widely, doing greater outreach, and broadening the delivery of the program beyond NSPs and sexual health to liver clinics and other settings. Further, as one Non-Aboriginal or Torres Strait Islander worker noted, ‘the potential would be taking the existing model as it works and trying to increase that geographically to the areas where that’s not happening at the moment.’

And every community is different, every mob is different, what works in one might not work in another, but it definitely has to be more flexible. (Aboriginal or Torres Strait Islander DLM worker)

I think DLM should be broadened.… I don’t DLM should just be for NSP. I think scaling up of DLM should be across liver clinics, NSP and sexual health services. I think you would get a lot greater compliance with education, testing and diagnosis and treatment if it was across all three. (Non-Aboriginal or Torres Strait Islander DLM worker)

Beyond BBVs and STIs, staff suggested that the DLM model could be broadened to address a range of chronic health conditions that disproportionately affect Aboriginal and Torres Strait Islander communities. In some instances, the DLM model was used to inform the development of other programs run by the sites. This has allowed for the continuation of the intervention, albeit not necessarily in the original form it was intended. For instance, one site used learnings from DLM to inform the development of a broader intervention for Aboriginal and Torres Strait Islander clients on a range of health issues. Some of the learnings from DLM included having a designated Aboriginal or Torres Strait Islander worker, how or where clients would be recruited from, and how clients could be followed up over time. This should not be viewed as a failure of the program, but rather show the potential for an intervention such as this to be scaled up and implemented to address a broader range of health concerns.

[W]e ripped off the methodology, thank you very much, with a view to looking at broader issues around diabetes, smoking and anxiety (Non-Aboriginal or Torres Strait Islander DLM worker)

I guess you could also adapt and apply it for many different things. You know, obviously at the moment it’s STI’s, BBI centred, but you could have exactly the same model for diabetes, you know chronic care, other chronic care issues like heart disease, smoking, those sorts of things, there’s no reason it has to stay just within that small realm of BBI’s and STI’s, especially since we know unfortunately so many of the clients who we see here, have got many complex comorbidities in health. (Non-Aboriginal or Torres Strait Islander DLM worker)

## Conclusion

This paper uses DLM staff and client insights to reflect on how the DLM program applies to each of the dimensions of the RE-AIM framework for interventions. RE-AIM is a useful framework when examining interventions, given that it provides the opportunity to examine how the intervention was intended to be implemented against the messy, real-world implementation of programs. Our findings illustrate this point in relation to mainstream NSP and sexual health services. The DLM program was originally designed to be run at two pilot sites, with the NSP and sexual health services closely co-located, to facilitate referral pathways for BBV and STI screening and treatment. However, when scaled up and expanded across several new sites, our findings highlight the need for adaptability and flexibility of the program to suit the local context and community needs. For instance, new sites did not necessarily have co-located NSP and sexual health services and required solutions (such as transporting clients) to this disjoint between education and screening in order to ensure a smooth referral pathway. In another example, new sites were faced with challenges like travel distances (in regional/rural areas) and parking difficulties, resulting in the site running outreach programs and altering the incentive payment amounts provided to clients to try to encourage clients to return for screenings, results, and vaccination or STI treatment. Sites also faced other challenges such as ensuring clients returned to progress through the program, such as to receive their results. This challenge may be resolved in future with the development of effective point-of-care testing for BBVs and STIs (see [49]).

Despite the challenges of scaling up and implementing the program, the findings documented in this paper highlight the numerous benefits of the DLM program in facilitating relationships between Aboriginal and Torres Strait Islander people and mainstream health services. Having frontline Aboriginal and Torres Strait Islander workers was seen by staff and clients to be critical in creating a culturally appropriate and safe environment and attempting to rebuild trust in mainstream health systems. The initial introduction by Aboriginal and Torres Strait Islander workers was perceived as engendering positive first interactions with mainstream health services (including with non-Aboriginal and Torres Strait Islander health staff), which could encourage clients to return for repeat health interactions in future beyond engagement in the program. Further, incentivising engagement in a health promotion program may be considered to be controversial for some, however, our data show that such incentives are effective in at least getting clients to first attend the service. It is the relationships built with health workers, however, which sustain their attendance over and above these incentives. As we have demonstrated here and elsewhere [24, 31], the DLM program is effective in achieving its stated aims of increasing engagement of Aboriginal and Torres Strait Islander people in viral hepatitis education, and screening and treatment for BBVs and STIs. The long-term sustainability of models like DLM must be ensured, including the funding of identified Aboriginal and Torres Strait Islander positions, even where DLM has been subsumed within other programs run by these health services.

This study is limited by the self-selecting sample of DLM clients and staff. Originally, we intended to recruit a range of clients from the service, including those who had dropped off at various stages of the DLM program or those who refused to participate in the program. Ultimately, we were unable to engage these clients, and thus their perspectives were not included in this research. Future iterations of the program and evaluation should seek to obtain broader perspectives of the program to examine barriers to engagement in DLM and opportunities for improvement.

By documenting the challenges (and positive outcomes) from implementing the DLM program, we hope to share the learnings from these nine sites with others who may be considering implementation of DLM or other similar programs targeted at Aboriginal and Torres Strait Islander communities. To further assist those who may be interested in implementing DLM, we have developed an implementation toolkit with further resources and templates to guide this process (see https://www.deadlylivermob.org). The findings from this paper and others [25, 31, 50] show that this program has the potential to be a ‘one stop shop’ for concerns around BBVs and STIs, but also that the model has the potential to be adapted and expanded to speak to some of the systemic inequalities within health systems by addressing a broader range of health concerns that disproportionately impact Aboriginal and Torres Strait Islander Australians.

## Data Availability

The datasets generated and/or analysed during the current study are not publicly available due to the possibility of individual privacy being compromised.

## Abbreviations

BBV: Blood borne virus
DAAs: Direct-acting antiviral
DLM: Deadly Liver Mob
HARP: HIV & Related Programs
HAV: Hepatitis A
HBV: Hepatitis B
HCV: Hepatitis C
LHD: Local Health District
NSP: Needle and Syringe Program
NSW: New South Wales
SIC: Safe Injecting Cwiz
STI: Sexually transmissible infection

## Footnotes

1 This figure is based on the 2018 report as the 2021 report is based on small numbers. 2021 and 2022 reports suggest that HIV notifications are similar among Aboriginal and Torres Strait Islander and non-Aboriginal or Torres Strait Islander Australians, however these data are based on small numbers and the report suggests may not be reflective of national trends (Kirby Institute, 2021, 2022).

2 Christie (2010b, p. 40) describes that ‘effective health literacy is largely to do with effective communication’. Thus, we take the view that health literacy among First Nations communities must not be understood in terms of individual deficits, but instead as whether effective communication exists between health systems and communities (i.e., as a health systems issue).

3 LHDs manage any public hospitals and health services within defined geographical areas in NSW. There are currently 15 LHDs across the state; eight covering the Sydney metropolitan area and seven covering rural and regional NSW (see https://www.health.nsw.gov.au/lhd/Pages/default.aspx).
