# Correction: Insights from the scale-up and implementation of the Deadly Liver Mob program across nine sites in New South Wales, Australia, according to the RE-AIM framework

**DOI:** 10.1186/s12954-024-00926-x

**Published:** 2024-02-06

**Authors:** Elena Cama, Kim Beadman, Mitch Beadman, Melinda Walker, Carla Treloar

**Affiliations:** Centre for Social Research in Health, John Goodsell Building, UNSW Sydney, Sydney, NSW 2052 Australia

## Correction: Harm Reduction Journal (2023) 20:154 10.1186/s12954-023-00889-5

Following publication of the original article [1], the reference 25 has been added to the reference list and the same has been shown below:

25. Treloar, C., Beadman, K., Beadman, M. et al. Evaluating a complex health promotion program to reduce hepatitis C among Aboriginal and Torres Strait Islander peoples in New South Wales, Australia: the Deadly Liver Mob. Harm Reduct J 20, 153 (2023). 10.1186/s12954-023-00885-9

The original article has been corrected.
